# Management Challenges of Psychosis and Aggression Secondary to Traumatic Brain Injury: A Report of Two Cases

**DOI:** 10.7759/cureus.86474

**Published:** 2025-06-21

**Authors:** Jude Beauchamp, Tania Sultana, Amir Meftah, Satwant Singh, Chino Ezema, Sana Elham Kazi, Muhammad Azam, Jacky S Petion, Bamidele O Johnson, Esther U Ezenagu, Bashir Aribisala, Thant Htet, Patrice Fouron, Jeffery Lawrence, Tolu Olupona

**Affiliations:** 1 Psychiatry and Behavioral Sciences, One Brooklyn Health – Interfaith Medical Center, New York, USA; 2 Psychiatry, One Brooklyn Health – Interfaith Medical Center, New York, USA

**Keywords:** aggressive behavior, antipsychotic medications, cognition impairment, psychosis, traumatic brain injury

## Abstract

Millions of people in the United States suffer from traumatic brain injury (TBI) yearly. Individuals recovering from moderate to severe TBI are at risk of developing medical and psychiatric comorbidities. Psychosis due to TBI appears to be an infrequent yet serious complication. Psychosis secondary to TBI is debilitating, and its management remains challenging. Individuals may have complex clinical presentations, such as behavioral disturbances (impulsivity or aggression) or other comorbid conditions (anxiety, depression, PTSD (post-traumatic stress disorder), substance use disorders, and seizure disorders). Atypical antipsychotics are the first line of treatment, along with psychotherapy. Mood stabilizers or antidepressants should be considered for mood disturbance. Other comorbid conditions must be addressed promptly to improve outcomes and stabilize the patient in the community. In this article, we discuss two cases that developed psychosis secondary to TBI along with comorbid conditions and their management.

## Introduction

Traumatic brain injury (TBI) is brain damage or alteration of brain function due to external forces. TBI is clinically classified by severity into mild, moderate, and severe based on the state of consciousness, amnesia, and other neurological symptoms [1]. TBI pathophysiology involves two types of damage: primary and secondary. Primary damage is produced by external force, which includes increased intracranial pressure, hemorrhage, edema, stretching, and tearing of axons, leading to diffuse axonal injury (DAI) and direct intracellular damage [1,2]. Secondary damage refers to cellular and molecular injury, which includes further axonal degeneration and demyelination, oxidative stress, neuroinflammation, and mitochondrial and synaptic damage. Secondary damage occurs hours to days after the TBI and is responsible for the persistence of symptoms and other neurodegenerative disorders [2,3].

Moderate to severe TBI can cause personality changes such as impulsivity, severe irritability, and affective instability [4]. All TBIs are associated with a range of affective symptoms, suicidality, and worsening or new-onset psychiatric disorders, including mood disorders, anxiety disorders, cognitive disorders, psychotic disorders, changes in personality and behavior, and sleep disorders, among others [4]. Psychosis is an infrequent but potentially debilitating consequence of TBI. The role of TBI in the development of psychosis is complex, and the management remains challenging [5]. In this article, we present two cases that developed psychosis after TBI.

## Case presentation

Case presentation 1

A 44-year-old African American male with an unclear past psychiatric history and a past medical history of TBI two years ago presented with aggressive behavior, thoughts of hurting others, and disorganized behavior. On evaluation, the patient endorsed paranoid delusions that the hospitals he had been admitted to in the past had stolen his brain, that people were taking his money, and that individuals conspiring with hospitals were stealing from him. The patient perseverated on his admission to three different hospitals for post-TBI care. He stated that the brain injury changed him. He was unable to elaborate despite several attempts by the team. According to the record, the onset of psychiatric symptoms coincided with the brain injury. He also endorsed auditory hallucinations. A delusion of grandeur was elicited; the patient stated he had superpowers like Spider-Man. The patient has a history of substance use (alcohol and cannabis), and urine toxicology was positive for cannabis during his presentation. He also has a history of thyroid nodules and surgery (1999) and knee surgery due to a ligament injury (2016).

**Figure 1:**
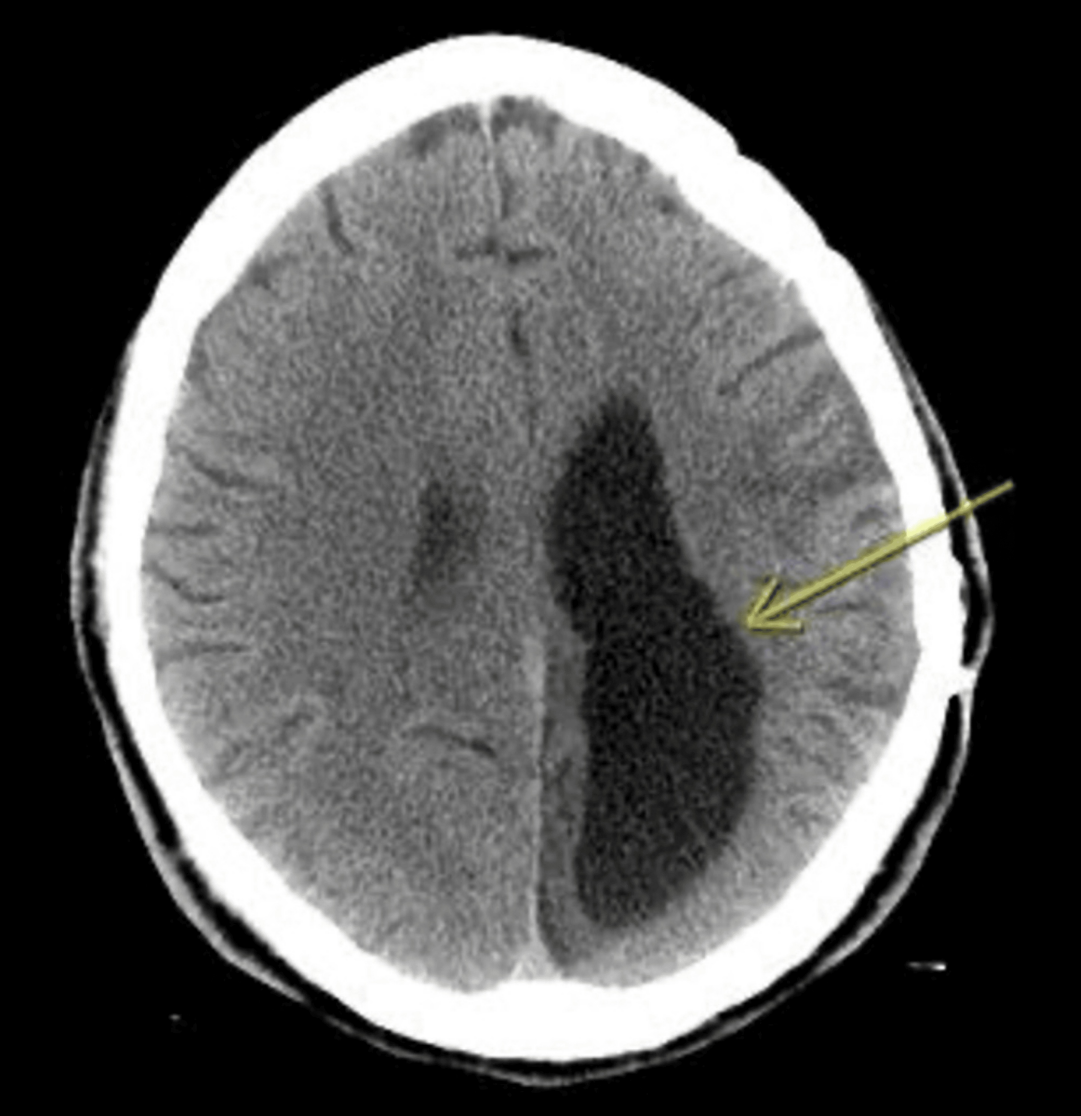
CT brain without contrast showed left frontal and temporal encephalomalacia and left occipital parietal chronic infraction.

**Figure 2:**
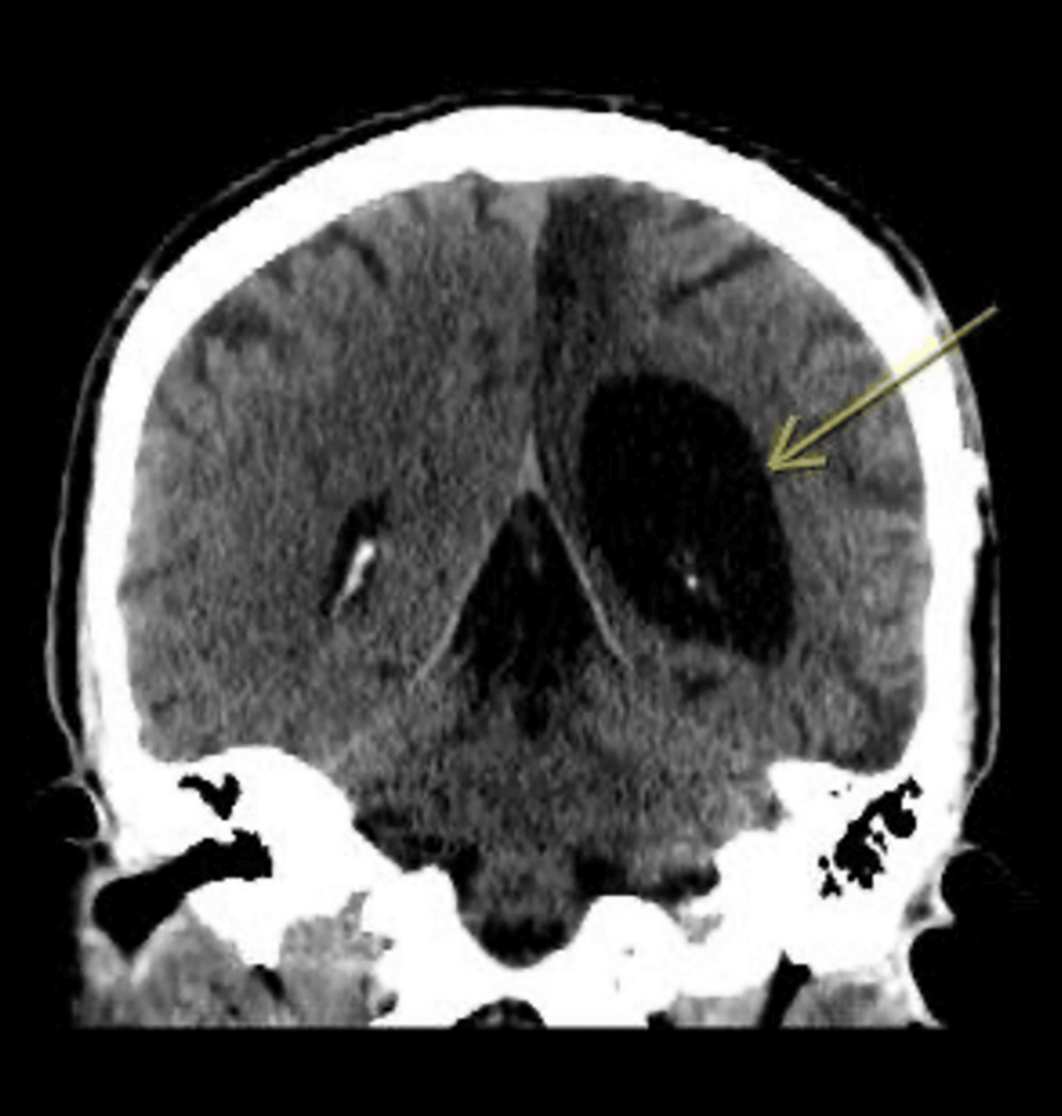
CT brain without contrast showed left frontal and temporal encephalomalacia and left occipital parietal chronic infraction.

The patient has a history of massive TBI with hemorrhage and a history of craniotomy (2020), two years prior to his presentation at our hospital. He had a history of assault resulting in cerebrovascular accident, left subdural hematoma requiring left craniotomy, bilateral subarachnoid hemorrhage, and left posterior cerebral artery infarct. The treatment team could not locate the previous CT scan report. A CT scan of the head without contrast was performed during admission. Findings included left-sided craniotomy changes with left frontal and temporal encephalomalacia and paramedian left occipital-parietal chronic infarction (Figures 1, 2). No acute hemorrhage, acute infarction, or mass effect was identified. 

The urine toxicology report was positive for cannabis and ethanol. It was negative for other illicit drugs, including cocaine, barbiturates, methadone, amphetamines, PCP (phencyclidine), and opiates. His blood alcohol content was elevated at 22.85 mg/dL. His TSH (thyroid-stimulating hormone) level was low, i.e., 0.357 uIU/mL, given his past medical history of thyroid nodule and surgical history.

Aripiprazole 10 mg was started to address psychosis. The patient was non-compliant with oral medications during hospitalization due to poor insight. Therefore, he received Abilify Maintena 400 mg IM for psychosis. He also received IM PRN (pro re nata) medication for aggressive behavior, which included Thorazine 100 mg IM and Ativan 2 mg IM. Over the hospital course, the patient showed improvement in his symptoms. He received sessions of supportive psychotherapy and group therapy. Over the course of the hospital stay, the patient’s psychotic symptoms improved. He denied homicidal ideation. The patient was subsequently discharged to a TBI facility.

Case presentation 2

The patient is a 51-year-old African American male with a past psychiatric history of unspecified psychosis and a past medical history of seizure disorder and encephalopathy, unspecified. At age 18, the patient was involved in a shooting that left him with a focal traumatic brain injury. Prior to the event, the patient did not have any past psychiatric history or prior psychiatric hospitalization. Per his mother, the patient started to develop a seizure disorder, unprovoked anger outbursts, and, a couple of years after the TBI event, he was subsequently hospitalized several times. 

**Figure 3:**
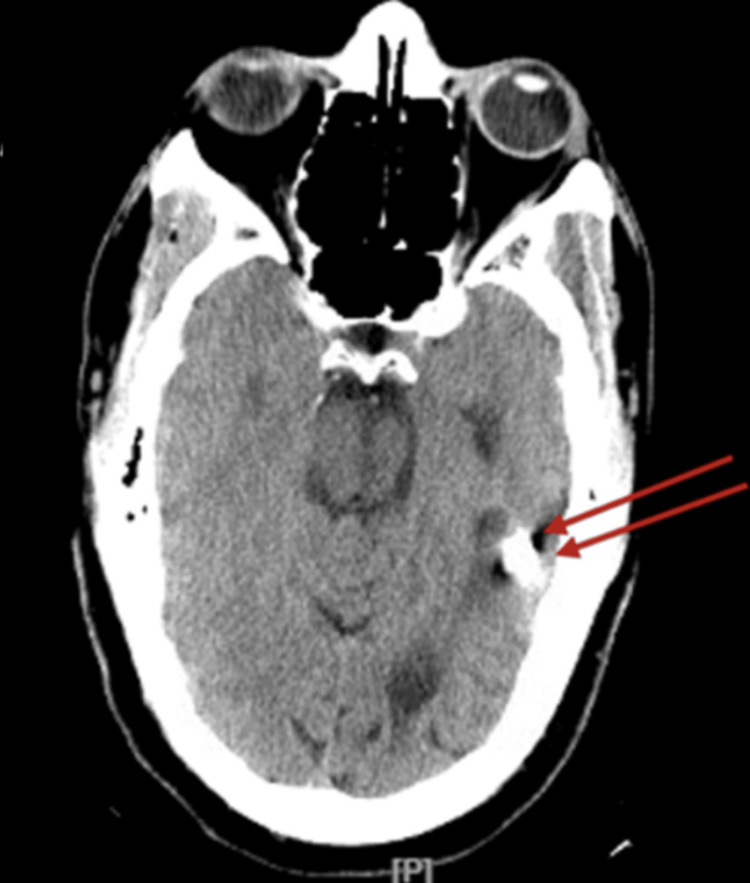
Head CT scan, without contrast. A metallic bullet fragment is identified in the left temporoparietal region from a prior gunshot wound. Basal ganglia old infarct with gliosis.

**Figure 4:**
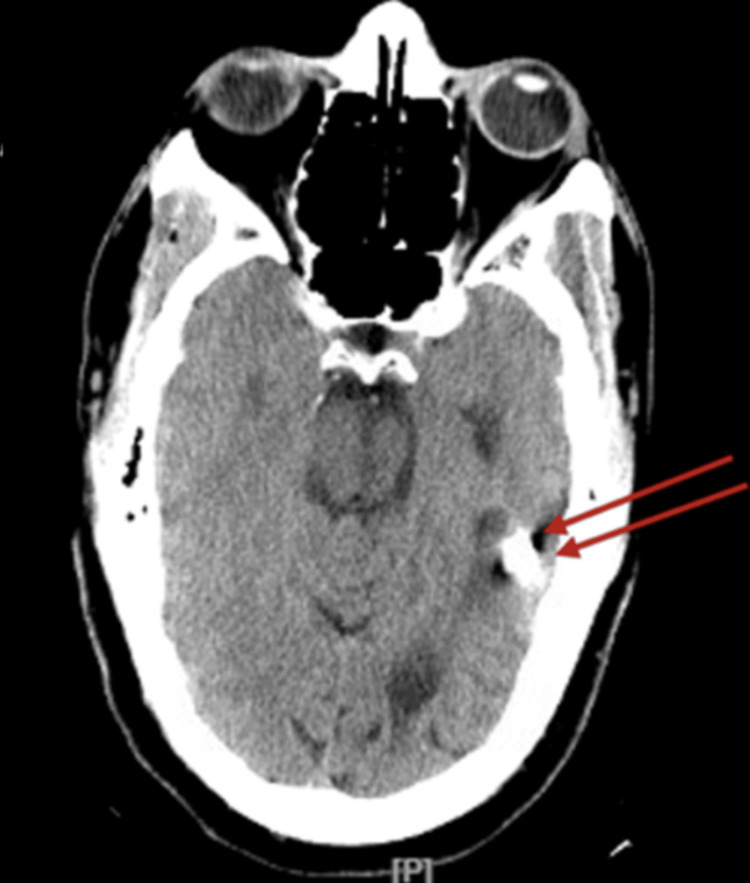
Head CT scan, coronal view. Metallic fragments are identified in the right frontal lobe, probably from a prior gunshot wound. There are operative changes of right frontal craniotomy.

From the patient’s medical record review, a computerized scan (CT scan) of the brain without contrast done in 2010 reported a 15 mm bullet fragment in the left temporal lobe, stable in position from prior study; an additional 4 mm ballistic fragment in the right frontal lobe; and a stable appearance of encephalomalacia along the ballistic track transecting the bilateral frontal lobes. There was mild enlargement of the ventricular system and cortical sulci consistent with parenchymal volume loss. The original CT scan images could not be found. In comparison, the patient had another CT scan without contrast done in September 2023 that demonstrated the metallic bullet fragment in the left temporoparietal region from the previously reported gunshot wound (Figure 3). Metallic fragments were also identified in the right frontal lobe, probably from the prior gunshot wound (Figure 4). Left temporal lobe and basal ganglia old infarct with gliosis. No acute intracranial abnormalities were found (Figures 3, 4).

From medical record reviews, the patient’s first psychotic episode was in 2021, when he was admitted for unspecified schizophrenia spectrum and other psychotic disorders. The patient was diagnosed the same year with adjustment disorder with depressed mood. The patient has several documented hospitalizations for unspecified seizures in the same year as well. In 2022, the patient was admitted for another psychotic episode due to aggressive behavior and paranoid delusions. 

A thorough review of the patient’s past medical, social, and family history failed to identify contributing factors other than the TBI. There is no family psychiatric history. The patient denied a history of substance use. Per the medical record review, the patient’s urine toxicology reports over the last two years were negative for illicit drugs, including cannabis, cocaine, barbiturates, methadone, amphetamines, PCP, and opiates. There is no history of physical trauma besides the TBI event in 2010. 

In 2023, the patient was brought to the Psychiatric Emergency Department (Psych ED) by Emergency Medical Services (EMS), activated by the nursing home staff where the patient lives, for aggressive and paranoid behavior. Per the EMS report, the patient exhibited paranoid behavior, barricading himself in the nursing home dining area, and was preoccupied with sex. On the day of admission to the ED, the patient continued to display hypersexualized and aggressive behavior. The patient attacked staff and was managed with 2 mg lorazepam and 5 mg haloperidol. Later that day, the patient was reassessed and found less agitated, but the agitation appeared to be triggered when asked about medication. The patient was admitted to the inpatient unit for continuation of care. On the unit, the patient was placed on PO levetiracetam 750 mg BID, PO mirtazapine 15 mg at bedtime, and PO olanzapine 10 mg at bedtime. However, the patient declined to take any medication. Following continued psychoeducation, the patient eventually agreed to take a long-acting injectable (LAI), Haldol Decanoate 100 mg. Over time, the patient’s psychotic symptoms improved on the unit. The management team continued to provide milieu therapy in addition to the monthly LAI injection while the patient awaited safe discharge to a nursing home.

## Discussion

TBI can be associated with a wide range of psychiatric and behavioral conditions [2]. Patients with TBI appear to develop psychotic symptoms more frequently than the general population. The onset of psychotic symptoms can be early or late [6]. Management may involve pharmacotherapy with atypical antipsychotics as the first-line treatment [4,6], along with non-pharmacological interventions such as electroconvulsive therapy, music therapy, behavioral strategies, and environmental modifications. These may include identifying and removing triggers, or using methods such as redirection, distraction, and time-out [7]. Atypical antipsychotics (e.g., risperidone, olanzapine, quetiapine, ziprasidone, and clozapine) have demonstrated greater efficacy, particularly in reducing both negative and positive symptoms, and in decreasing agitation and aggression. Clozapine is considered the most effective atypical antipsychotic agent; however, it carries a greater risk of causing seizures, which is an important consideration when treating patients with a history of TBI [8]. Antipsychotic monotherapy should be prioritized due to the increased risk of side effects associated with polypharmacy [9]. However, clinical experience shows that a single agent may not always result in satisfactory symptomatic improvement [8]. In conjunction with antipsychotics, mood stabilizers such as valproic acid, lithium, and antidepressant medications are commonly used [6]. Non-invasive brain stimulation, such as repetitive transcranial magnetic stimulation (rTMS), can be an effective treatment option for post-TBI symptoms, including depression, tinnitus, and neglect, although the safety of this method remains uncertain [10]. rTMS has been shown to reduce the severity of musical auditory hallucinations that developed following a right temporal lobe injury [10,11]. Following treatment, a PET scan indicated that rTMS reduced brain activity in the right temporal region [11].

Both cases were managed with LAI, and their symptoms improved after a long inpatient hospitalization. In addition to LAI, they received PRN medications for agitation. Alongside pharmacotherapy, patients received supportive and group therapy. Case 1 was treated with an atypical antipsychotic, and case 2 was treated with a typical antipsychotic. Concomitant medications were administered to address comorbid conditions. Patients who develop psychosis secondary to TBI may occasionally exhibit aggression [12].

Close monitoring and ongoing assessment of the patient’s symptoms and response to treatment are essential to optimize long-term outcomes [13]. By recognizing the association between TBI and psychosis and conducting thorough evaluations, clinicians can provide optimal care for individuals at risk of developing psychosis following a TBI. In both cases, the TBI event appears to have contributed to the development of the patient’s psychotic disorder.

## Conclusions

TBI appears to be a common outcome, with millions of people exposed each year. Patients with moderate to severe TBI are at greater risk of developing TBI-related psychosis. Psychosis following TBI itself appears uncommon; however, its consequences can be debilitating, with challenging and complex management. TBI can increase the risk of developing psychosis and behavioral disturbances, which may present as primary psychotic disorders. Clinicians should carefully review a patient’s medical and psychiatric history. We found that both typical and atypical antipsychotics, given separately or in combination, appear to be beneficial in managing psychosis in patients with TBI.
